# Supplementary material for: Interconnection of Gut Microbiome and Efficacy of Immune Checkpoint Inhibitors in Inoperable Non-Small-Cell Lung Cancer
Source: Int J Mol Sci. 2025 Aug 11;26(16):7758. doi: 10.3390/ijms26167758 (PMC12386741; doi:10.3390/ijms26167758)
Supplement: Supplementary file 1 [file ijms-26-07758-s001.zip › ijms-3782503-supplementary.pdf]

Table S1. Comparison of Chao1 index values for patients cohorts startified by the clinical characteristics - progression, benefit from IO therapy, age, progression free survival (PFS), overall survival (OS). MW_P-value - Mann-Whitney two sided test p-value. For quantitative characteristics, "Yes" means that the patient cohort had the characteristics value more than or equal the threshold (Q1, Q2, or Q3 of the sample). Cells with p-value<0.05 are filled with green colour.

Characteristics	Total Read Depth	Yes_median	No_median	MW_P-value
Progression	1112	149.455	144.833	0.288
Progression	1112	144.440	131.830	0.170
Progression	1112	144.792	140.422	0.242
Progression	1112	143.333	142.636	0.237
Progression	1112	143.333	142.670	0.424
Progression	1112	144.063	140.400	0.200
Progression	1112	145.636	143.607	0.338
Progression	1112	153.118	135.876	0.192
Progression	1112	152.056	147.125	0.354
Progression	1112	146.050	134.327	0.258
Progression	2223	163.333	142.500	0.149
Progression	2223	166.000	145.528	0.124
Progression	2223	165.353	141.500	0.156
Progression	2223	165.250	144.625	0.200
Progression	2223	167.286	158.650	0.274
Progression	2223	170.500	138.692	0.231
Progression	2223	158.167	140.636	0.220
Progression	2223	184.154	153.520	0.136
Progression	2223	175.455	143.300	0.105
Progression	2223	159.517	142.303	0.216
Progression	3334	170.724	142.398	0.161
Progression	3334	171.474	144.110	0.233
Progression	3334	176.000	146.385	0.121
Progression	3334	179.231	144.236	0.080
Progression	3334	163.619	153.000	0.156
Progression	3334	179.786	152.806	0.103
Progression	3334	194.292	148.333	0.091
Progression	3334	180.000	142.594	0.127
Progression	3334	167.714	143.400	0.146
Progression	3334	187.786	145.000	0.156
Progression	4445	186.261	152.250	0.136
Progression	4445	183.714	145.107	0.212
Progression	4445	184.889	145.450	0.177
Progression	4445	180.913	139.955	0.124
Progression	4445	185.800	148.000	0.156
Progression	4445	191.103	151.333	0.133
Progression	4445	194.333	143.814	0.123
Progression	4445	194.136	141.750	0.124
Progression	4445	179.731	149.406	0.208
Progression	4445	170.143	144.927	0.139
Progression	5556	198.531	151.607	0.117
Progression	5556	195.640	149.385	0.127
Progression	5556	197.150	147.176	0.123
Progression	5556	198.207	151.115	0.136
Progression	5556	190.577	144.038	0.082

Progression	5556	199.476	148.167	0.126
Progression	5556	188.154	146.663	0.129
Progression	5556	188.875	144.237	0.139
Progression	5556	191.038	152.931	0.177
Progression	5556	191.097	156.077	0.157
Progression	6667	199.240	145.600	0.083
Progression	6667	193.600	146.300	0.066
Progression	6667	199.833	148.167	0.105
Progression	6667	203.731	154.875	0.132
Progression	6667	195.217	144.500	0.119
Progression	6667	198.682	148.750	0.166
Progression	6667	200.125	148.875	0.130
Progression	6667	195.545	144.700	0.144
Progression	6667	191.517	149.500	0.116
Progression	6667	184.120	153.464	0.085
Progression	7778	207.952	153.750	0.175
Progression	7778	199.875	147.231	0.157
Progression	7778	197.778	156.000	0.139
Progression	7778	198.183	161.333	0.171
Progression	7778	195.523	152.667	0.181
Progression	7778	203.136	146.000	0.138
Progression	7778	202.000	151.750	0.161
Progression	7778	198.250	146.000	0.171
Progression	7778	206.685	146.000	0.123
Progression	7778	208.342	146.231	0.135
Progression	8889	215.500	185.154	0.257
Progression	8889	205.077	189.077	0.208
Progression	8889	203.542	183.000	0.233
Progression	8889	211.500	178.188	0.202
Progression	8889	202.840	186.583	0.262
Progression	8889	209.533	188.000	0.210
Progression	8889	210.286	192.000	0.235
Progression	8889	212.000	185.500	0.231
Progression	8889	216.182	189.077	0.244
Progression	8889	212.000	182.100	0.229
Progression	10000	204.350	188.642	0.229
Progression	10000	212.876	191.419	0.159
Progression	10000	217.700	195.306	0.206
Progression	10000	220.794	183.898	0.196
Progression	10000	208.142	182.420	0.188
Progression	10000	216.156	191.998	0.181
Progression	10000	211.804	191.808	0.149
Progression	10000	212.873	188.833	0.173
Progression	10000	210.633	187.455	0.164
Progression	10000	205.613	191.950	0.192
Benefit	1112	169.363	133.231	0.006
Benefit	1112	157.543	127.353	0.029
Benefit	1112	153.875	127.929	0.023
Benefit	1112	149.281	130.136	0.049
Benefit	1112	172.832	129.400	0.007
Benefit	1112	156.210	131.750	0.036
Benefit	1112	169.490	121.368	0.002

Benefit	1112	155.158	125.071	0.017
Benefit	1112	165.692	123.071	0.014
Benefit	1112	164.938	135.286	0.016
Benefit	2223	170.732	136.750	0.013
Benefit	2223	179.027	147.063	0.013
Benefit	2223	185.337	137.615	0.025
Benefit	2223	179.497	143.400	0.019
Benefit	2223	194.130	136.071	0.002
Benefit	2223	190.466	136.000	0.017
Benefit	2223	186.500	133.300	0.019
Benefit	2223	207.694	146.800	0.007
Benefit	2223	183.818	144.375	0.018
Benefit	2223	175.958	136.773	0.018
Benefit	3334	192.438	141.040	0.009
Benefit	3334	189.222	148.375	0.013
Benefit	3334	195.897	146.769	0.012
Benefit	3334	197.963	142.043	0.014
Benefit	3334	186.859	154.037	0.012
Benefit	3334	212.674	141.077	0.008
Benefit	3334	198.518	149.000	0.013
Benefit	3334	197.978	143.400	0.013
Benefit	3334	193.688	151.565	0.008
Benefit	3334	198.827	140.000	0.016
Benefit	4445	203.500	139.000	0.004
Benefit	4445	202.347	143.043	0.007
Benefit	4445	202.094	143.400	0.016
Benefit	4445	209.469	149.542	0.006
Benefit	4445	207.411	150.000	0.007
Benefit	4445	215.558	151.056	0.007
Benefit	4445	210.334	144.542	0.006
Benefit	4445	204.366	151.063	0.013
Benefit	4445	202.357	149.813	0.006
Benefit	4445	213.589	151.750	0.010
Benefit	5556	215.040	144.077	0.005
Benefit	5556	212.250	152.769	0.012
Benefit	5556	215.639	154.250	0.007
Benefit	5556	207.607	149.462	0.006
Benefit	5556	208.186	142.077	0.006
Benefit	5556	206.958	155.750	0.014
Benefit	5556	220.065	147.077	0.008
Benefit	5556	215.773	153.130	0.004
Benefit	5556	207.500	148.462	0.006
Benefit	5556	216.940	139.125	0.002
Benefit	6667	213.200	165.476	0.008
Benefit	6667	208.657	165.167	0.004
Benefit	6667	210.423	144.120	0.005
Benefit	6667	219.109	141.214	0.004
Benefit	6667	212.630	148.773	0.005
Benefit	6667	207.552	154.556	0.015
Benefit	6667	217.600	159.250	0.006
Benefit	6667	208.501	143.500	0.005
Benefit	6667	216.920	149.773	0.004

Benefit	6667	220.980	150.500	0.003
Benefit	7778	216.700	157.029	0.005
Benefit	7778	226.885	161.477	0.006
Benefit	7778	222.714	160.794	0.006
Benefit	7778	229.286	157.636	0.006
Benefit	7778	222.107	157.051	0.006
Benefit	7778	229.724	155.725	0.007
Benefit	7778	219.500	158.401	0.009
Benefit	7778	215.000	150.063	0.006
Benefit	7778	228.500	154.132	0.005
Benefit	7778	224.957	153.681	0.009
Benefit	8889	233.750	174.000	0.021
Benefit	8889	233.500	174.000	0.017
Benefit	8889	234.885	174.000	0.021
Benefit	8889	237.391	174.000	0.022
Benefit	8889	230.714	174.000	0.018
Benefit	8889	238.000	174.000	0.020
Benefit	8889	233.538	174.000	0.012
Benefit	8889	232.241	174.000	0.020
Benefit	8889	230.600	174.000	0.024
Benefit	8889	239.526	174.000	0.018
Benefit	10000	237.167	183.909	0.068
Benefit	10000	225.000	178.125	0.109
Benefit	10000	233.120	178.000	0.113
Benefit	10000	233.966	183.545	0.079
Benefit	10000	240.550	182.714	0.104
Benefit	10000	235.000	174.652	0.086
Benefit	10000	231.600	184.750	0.108
Benefit	10000	235.273	184.000	0.102
Benefit	10000	236.059	180.125	0.090
Benefit	10000	234.091	183.250	0.104
Age Q1	1112	152.667	122.275	0.182
Age Q1	1112	153.000	124.857	0.084
Age Q1	1112	149.000	128.279	0.135
Age Q1	1112	146.063	129.324	0.064
Age Q1	1112	147.273	128.942	0.182
Age Q1	1112	152.545	128.954	0.081
Age Q1	1112	153.875	126.219	0.102
Age Q1	1112	153.118	130.057	0.129
Age Q1	1112	158.250	121.315	0.178
Age Q1	1112	157.276	130.334	0.201
Age Q1	2223	164.484	131.675	0.123
Age Q1	2223	166.000	131.275	0.102
Age Q1	2223	165.353	130.913	0.143
Age Q1	2223	170.545	125.885	0.070
Age Q1	2223	172.500	125.938	0.066
Age Q1	2223	176.118	129.308	0.103
Age Q1	2223	170.000	130.885	0.074
Age Q1	2223	178.583	129.042	0.108
Age Q1	2223	166.600	133.295	0.091
Age Q1	2223	161.545	130.851	0.145
Age Q1	3334	173.429	133.167	0.114

Age Q1	3334	171.474	132.813	0.147
Age Q1	3334	175.000	130.900	0.094
Age Q1	3334	179.231	130.682	0.195
Age Q1	3334	173.750	129.833	0.166
Age Q1	3334	173.071	129.656	0.143
Age Q1	3334	179.813	133.527	0.070
Age Q1	3334	179.000	131.214	0.070
Age Q1	3334	167.714	131.667	0.162
Age Q1	3334	174.500	133.467	0.126
Age Q1	4445	178.182	132.288	0.147
Age Q1	4445	172.429	131.250	0.154
Age Q1	4445	174.111	132.100	0.100
Age Q1	4445	180.913	130.238	0.125
Age Q1	4445	178.500	130.988	0.074
Age Q1	4445	175.353	131.000	0.114
Age Q1	4445	187.059	132.000	0.125
Age Q1	4445	177.714	131.958	0.133
Age Q1	4445	174.500	132.208	0.126
Age Q1	4445	175.000	132.833	0.117
Age Q1	5556	185.929	135.125	0.158
Age Q1	5556	183.000	132.125	0.099
Age Q1	5556	180.619	132.425	0.121
Age Q1	5556	176.714	132.500	0.117
Age Q1	5556	175.333	132.214	0.141
Age Q1	5556	179.000	132.800	0.125
Age Q1	5556	185.000	132.000	0.100
Age Q1	5556	179.875	132.333	0.082
Age Q1	5556	191.038	132.250	0.114
Age Q1	5556	181.000	133.417	0.125
Age Q1	6667	186.500	133.167	0.176
Age Q1	6667	181.895	133.321	0.152
Age Q1	6667	180.118	131.667	0.134
Age Q1	6667	187.120	132.667	0.117
Age Q1	6667	188.500	132.000	0.140
Age Q1	6667	179.667	132.500	0.095
Age Q1	6667	180.333	132.667	0.149
Age Q1	6667	182.929	131.500	0.100
Age Q1	6667	184.700	132.500	0.085
Age Q1	6667	184.120	132.500	0.123
Age Q1	7778	196.063	132.000	0.064
Age Q1	7778	189.125	132.500	0.038
Age Q1	7778	192.105	132.250	0.061
Age Q1	7778	194.588	132.500	0.043
Age Q1	7778	187.895	132.500	0.050
Age Q1	7778	185.571	132.667	0.088
Age Q1	7778	186.929	132.750	0.049
Age Q1	7778	190.895	132.500	0.056
Age Q1	7778	190.913	132.500	0.053
Age Q1	7778	205.091	132.000	0.033
Age Q1	8889	212.000	134.000	0.020
Age Q1	8889	211.056	134.000	0.030
Age Q1	8889	208.241	134.000	0.022

Age Q1	8889	211.500	134.000	0.016
Age Q1	8889	209.900	134.000	0.037
Age Q1	8889	209.533	134.000	0.035
Age Q1	8889	209.250	134.000	0.028
Age Q1	8889	212.000	134.000	0.024
Age Q1	8889	216.182	134.000	0.042
Age Q1	8889	212.000	134.000	0.029
Age Q1	10000	211.698	162.362	0.084
Age Q1	10000	214.615	165.019	0.072
Age Q1	10000	216.250	168.056	0.143
Age Q1	10000	218.063	161.875	0.131
Age Q1	10000	209.229	158.063	0.081
Age Q1	10000	216.772	165.685	0.111
Age Q1	10000	212.539	166.433	0.099
Age Q1	10000	212.560	163.500	0.099
Age Q1	10000	212.611	162.365	0.069
Age Q1	10000	214.271	165.565	0.072
PFS Q1	1112	152.667	135.115	0.213
PFS Q1	1112	145.000	133.874	0.222
PFS Q1	1112	149.056	126.295	0.199
PFS Q1	1112	146.063	131.841	0.208
PFS Q1	1112	147.273	129.918	0.180
PFS Q1	1112	145.500	135.762	0.385
PFS Q1	1112	153.875	125.448	0.086
PFS Q1	1112	153.118	127.952	0.259
PFS Q1	1112	154.000	124.430	0.172
PFS Q1	1112	158.333	135.862	0.199
PFS Q1	2223	163.333	137.250	0.261
PFS Q1	2223	166.000	148.147	0.215
PFS Q1	2223	171.667	138.620	0.315
PFS Q1	2223	173.250	149.309	0.272
PFS Q1	2223	172.500	136.875	0.182
PFS Q1	2223	180.619	145.188	0.312
PFS Q1	2223	174.500	139.150	0.154
PFS Q1	2223	166.077	158.714	0.326
PFS Q1	2223	167.538	148.717	0.272
PFS Q1	2223	160.500	139.439	0.145
PFS Q1	3334	178.577	145.097	0.199
PFS Q1	3334	176.250	149.214	0.210
PFS Q1	3334	180.500	154.985	0.231
PFS Q1	3334	179.231	145.593	0.199
PFS Q1	3334	177.045	157.626	0.190
PFS Q1	3334	179.786	152.066	0.222
PFS Q1	3334	179.813	149.609	0.256
PFS Q1	3334	179.000	148.836	0.236
PFS Q1	3334	181.773	151.640	0.174
PFS Q1	3334	174.500	142.458	0.254
PFS Q1	4445	184.158	144.217	0.140
PFS Q1	4445	190.036	144.093	0.180
PFS Q1	4445	189.556	151.475	0.241
PFS Q1	4445	183.833	151.271	0.166
PFS Q1	4445	187.188	150.528	0.182

PFS Q1	4445	181.773	153.528	0.147
PFS Q1	4445	182.053	158.571	0.246
PFS Q1	4445	184.217	155.481	0.241
PFS Q1	4445	181.435	159.515	0.190
PFS Q1	4445	185.028	158.029	0.204
PFS Q1	5556	185.929	152.775	0.162
PFS Q1	5556	194.714	155.218	0.227
PFS Q1	5556	188.565	156.520	0.174
PFS Q1	5556	198.207	152.626	0.158
PFS Q1	5556	177.036	158.038	0.174
PFS Q1	5556	190.300	160.232	0.174
PFS Q1	5556	185.000	160.538	0.174
PFS Q1	5556	186.652	163.315	0.140
PFS Q1	5556	191.500	156.043	0.186
PFS Q1	5556	191.097	149.938	0.133
PFS Q1	6667	193.000	165.501	0.195
PFS Q1	6667	193.600	170.583	0.180
PFS Q1	6667	181.400	149.854	0.162
PFS Q1	6667	187.120	154.557	0.176
PFS Q1	6667	189.500	156.439	0.172
PFS Q1	6667	194.538	157.153	0.249
PFS Q1	6667	200.125	160.560	0.170
PFS Q1	6667	190.300	149.000	0.158
PFS Q1	6667	186.500	154.161	0.158
PFS Q1	6667	198.136	154.521	0.138
PFS Q1	7778	207.000	157.029	0.076
PFS Q1	7778	197.750	161.477	0.084
PFS Q1	7778	196.000	160.794	0.081
PFS Q1	7778	200.750	157.636	0.072
PFS Q1	7778	197.545	157.051	0.067
PFS Q1	7778	199.000	155.725	0.068
PFS Q1	7778	201.500	158.401	0.096
PFS Q1	7778	199.955	150.063	0.060
PFS Q1	7778	206.500	154.132	0.058
PFS Q1	7778	205.091	153.681	0.092
PFS Q1	8889	212.000	174.000	0.434
PFS Q1	8889	211.056	174.000	0.500
PFS Q1	8889	208.241	174.000	0.462
PFS Q1	8889	213.286	174.000	0.497
PFS Q1	8889	217.037	174.000	0.447
PFS Q1	8889	209.533	174.000	0.465
PFS Q1	8889	210.286	174.000	0.447
PFS Q1	8889	214.500	174.000	0.484
PFS Q1	8889	216.182	174.000	0.484
PFS Q1	8889	213.773	174.000	0.484
PFS Q1	10000	213.135	174.000	0.282
PFS Q1	10000	216.600	174.000	0.293
PFS Q1	10000	217.000	174.000	0.400
PFS Q1	10000	219.538	174.000	0.298
PFS Q1	10000	216.158	174.000	0.349
PFS Q1	10000	219.000	174.000	0.326
PFS Q1	10000	213.034	174.000	0.367

PFS Q1	10000	213.625	174.000	0.344
PFS Q1	10000	214.000	174.000	0.332
PFS Q1	10000	222.000	174.000	0.293
OS Q1	1112	143.500	159.107	0.391
OS Q1	1112	140.500	149.741	0.459
OS Q1	1112	148.594	133.531	0.391
OS Q1	1112	143.333	139.773	0.453
OS Q1	1112	146.000	129.918	0.256
OS Q1	1112	144.063	144.131	0.434
OS Q1	1112	150.000	135.961	0.277
OS Q1	1112	142.000	146.028	0.406
OS Q1	1112	152.056	153.875	0.301
OS Q1	1112	140.154	141.558	0.388
OS Q1	2223	149.000	159.424	0.447
OS Q1	2223	163.077	167.024	0.431
OS Q1	2223	164.714	164.783	0.478
OS Q1	2223	164.333	162.898	0.440
OS Q1	2223	167.773	163.580	0.338
OS Q1	2223	158.615	167.952	0.459
OS Q1	2223	153.200	153.003	0.484
OS Q1	2223	166.077	163.506	0.379
OS Q1	2223	158.250	161.527	0.434
OS Q1	2223	154.391	166.033	0.290
OS Q1	3334	164.313	169.881	0.321
OS Q1	3334	163.750	171.276	0.326
OS Q1	3334	162.588	169.780	0.484
OS Q1	3334	153.040	167.310	0.344
OS Q1	3334	163.619	157.919	0.277
OS Q1	3334	164.000	168.854	0.321
OS Q1	3334	159.588	183.615	0.484
OS Q1	3334	168.955	176.557	0.385
OS Q1	3334	166.000	170.419	0.332
OS Q1	3334	159.000	180.380	0.481
OS Q1	4445	176.200	169.927	0.293
OS Q1	4445	170.240	172.214	0.341
OS Q1	4445	172.619	167.828	0.440
OS Q1	4445	168.667	173.767	0.332
OS Q1	4445	176.000	178.022	0.403
OS Q1	4445	175.353	174.500	0.338
OS Q1	4445	180.667	180.436	0.321
OS Q1	4445	177.714	168.509	0.440
OS Q1	4445	171.545	173.750	0.385
OS Q1	4445	170.143	167.820	0.391
OS Q1	5556	177.500	180.554	0.355
OS Q1	5556	177.000	178.889	0.428
OS Q1	5556	180.619	173.693	0.261
OS Q1	5556	176.714	174.027	0.332
OS Q1	5556	169.000	182.955	0.329
OS Q1	5556	179.000	169.768	0.385
OS Q1	5556	173.516	180.739	0.349
OS Q1	5556	174.474	180.200	0.277
OS Q1	5556	169.714	179.250	0.373

OS Q1	5556	177.368	177.047	0.272
OS Q1	6667	186.500	177.684	0.309
OS Q1	6667	179.882	183.488	0.335
OS Q1	6667	175.917	176.269	0.349
OS Q1	6667	179.231	180.052	0.335
OS Q1	6667	159.053	181.417	0.335
OS Q1	6667	170.333	186.508	0.437
OS Q1	6667	180.333	174.519	0.304
OS Q1	6667	181.625	182.278	0.344
OS Q1	6667	179.474	179.350	0.298
OS Q1	6667	181.375	177.727	0.264
OS Q1	7778	185.545	174.672	0.143
OS Q1	7778	181.138	182.313	0.178
OS Q1	7778	174.444	183.053	0.154
OS Q1	7778	183.182	184.294	0.186
OS Q1	7778	187.040	173.565	0.138
OS Q1	7778	185.571	178.091	0.143
OS Q1	7778	178.333	178.750	0.208
OS Q1	7778	190.895	178.947	0.186
OS Q1	7778	186.130	177.500	0.150
OS Q1	7778	194.063	191.571	0.188
OS Q1	8889	199.370	196.634	0.422
OS Q1	8889	201.583	197.913	0.425
OS Q1	8889	198.438	194.325	0.397
OS Q1	8889	202.788	197.625	0.422
OS Q1	8889	200.592	192.630	0.385
OS Q1	8889	199.554	203.518	0.379
OS Q1	8889	201.772	195.696	0.315
OS Q1	8889	203.313	184.629	0.416
OS Q1	8889	201.870	200.065	0.391
OS Q1	8889	207.969	192.346	0.344
OS Q1	10000	204.350	187.640	0.222
OS Q1	10000	214.418	198.013	0.213
OS Q1	10000	214.947	202.778	0.264
OS Q1	10000	219.519	202.560	0.154
OS Q1	10000	208.142	194.828	0.217
OS Q1	10000	216.156	191.802	0.272
OS Q1	10000	207.560	209.667	0.261
OS Q1	10000	211.827	198.500	0.272
OS Q1	10000	210.633	194.428	0.239
OS Q1	10000	205.613	197.183	0.236
Age Q2	1112	145.509	155.214	0.443
Age Q2	1112	142.470	141.429	0.363
Age Q2	1112	146.693	135.063	0.484
Age Q2	1112	142.531	143.333	0.318
Age Q2	1112	146.636	136.462	0.358
Age Q2	1112	149.023	135.300	0.384
Age Q2	1112	142.886	150.000	0.337
Age Q2	1112	151.612	136.105	0.363
Age Q2	1112	148.500	153.867	0.437
Age Q2	1112	139.859	146.050	0.345
Age Q2	2223	147.250	153.917	0.486

Age Q2	2223	156.538	168.030	0.456
Age Q2	2223	157.260	164.714	0.481
Age Q2	2223	157.955	164.333	0.481
Age Q2	2223	156.808	167.773	0.437
Age Q2	2223	147.341	176.118	0.348
Age Q2	2223	149.600	158.167	0.410
Age Q2	2223	157.181	183.731	0.437
Age Q2	2223	149.514	159.895	0.492
Age Q2	2223	152.696	159.517	0.500
Age Q2	3334	157.756	170.724	0.451
Age Q2	3334	147.663	171.474	0.371
Age Q2	3334	149.643	176.000	0.416
Age Q2	3334	150.583	177.500	0.426
Age Q2	3334	159.625	163.619	0.421
Age Q2	3334	147.326	170.120	0.323
Age Q2	3334	157.105	192.250	0.475
Age Q2	3334	147.719	184.000	0.432
Age Q2	3334	146.156	167.714	0.363
Age Q2	3334	154.000	186.261	0.497
Age Q2	4445	154.529	184.158	0.303
Age Q2	4445	148.563	183.714	0.340
Age Q2	4445	147.560	182.600	0.413
Age Q2	4445	157.885	180.913	0.386
Age Q2	4445	162.375	185.800	0.486
Age Q2	4445	165.964	181.773	0.437
Age Q2	4445	148.820	194.333	0.277
Age Q2	4445	152.185	196.682	0.353
Age Q2	4445	151.936	185.636	0.470
Age Q2	4445	151.250	170.143	0.384
Age Q2	5556	154.600	187.107	0.400
Age Q2	5556	154.885	195.640	0.405
Age Q2	5556	150.125	188.565	0.330
Age Q2	5556	153.764	200.690	0.313
Age Q2	5556	154.050	190.577	0.363
Age Q2	5556	152.243	190.300	0.360
Age Q2	5556	151.667	185.000	0.397
Age Q2	5556	151.107	186.652	0.340
Age Q2	5556	148.266	184.500	0.384
Age Q2	5556	152.794	191.097	0.345
Age Q2	6667	153.357	193.000	0.376
Age Q2	6667	158.682	194.500	0.325
Age Q2	6667	154.385	180.118	0.330
Age Q2	6667	154.750	187.120	0.286
Age Q2	6667	151.324	188.500	0.343
Age Q2	6667	154.327	198.682	0.323
Age Q2	6667	151.750	203.000	0.264
Age Q2	6667	156.824	190.556	0.429
Age Q2	6667	156.250	186.500	0.371
Age Q2	6667	159.500	184.120	0.337
Age Q2	7778	166.824	196.063	0.363
Age Q2	7778	165.563	189.125	0.320
Age Q2	7778	166.556	192.105	0.355

Age Q2	7778	168.545	194.588	0.308
Age Q2	7778	167.875	187.895	0.345
Age Q2	7778	165.577	192.545	0.421
Age Q2	7778	168.250	183.500	0.318
Age Q2	7778	164.200	197.000	0.308
Age Q2	7778	166.577	190.913	0.368
Age Q2	7778	165.618	205.091	0.303
Age Q2	8889	185.154	212.000	0.239
Age Q2	8889	189.077	205.077	0.203
Age Q2	8889	184.571	199.750	0.188
Age Q2	8889	178.188	203.750	0.199
Age Q2	8889	186.583	198.345	0.195
Age Q2	8889	188.000	209.533	0.195
Age Q2	8889	184.400	211.045	0.248
Age Q2	8889	185.500	194.625	0.184
Age Q2	8889	189.077	216.895	0.180
Age Q2	8889	182.100	203.938	0.197
Age Q2	10000	191.175	203.255	0.289
Age Q2	10000	201.257	209.881	0.189
Age Q2	10000	202.500	214.947	0.239
Age Q2	10000	197.773	220.775	0.340
Age Q2	10000	190.857	211.110	0.303
Age Q2	10000	200.313	209.052	0.261
Age Q2	10000	198.397	209.667	0.275
Age Q2	10000	198.393	218.267	0.280
Age Q2	10000	196.976	206.063	0.242
Age Q2	10000	192.500	205.063	0.211
PFS Q2	1112	169.363	133.231	0.004
PFS Q2	1112	157.543	127.353	0.017
PFS Q2	1112	153.875	127.929	0.019
PFS Q2	1112	149.281	130.136	0.045
PFS Q2	1112	172.832	130.619	0.007
PFS Q2	1112	156.210	131.750	0.033
PFS Q2	1112	169.490	129.792	0.003
PFS Q2	1112	155.158	125.071	0.014
PFS Q2	1112	165.692	123.071	0.010
PFS Q2	1112	164.938	135.286	0.018
PFS Q2	2223	170.732	136.750	0.013
PFS Q2	2223	179.027	147.063	0.012
PFS Q2	2223	185.337	137.615	0.019
PFS Q2	2223	179.497	143.400	0.015
PFS Q2	2223	194.130	136.071	0.002
PFS Q2	2223	190.466	136.000	0.014
PFS Q2	2223	186.500	133.300	0.015
PFS Q2	2223	207.694	146.800	0.006
PFS Q2	2223	183.818	144.375	0.016
PFS Q2	2223	175.958	136.773	0.015
PFS Q2	3334	192.438	141.040	0.007
PFS Q2	3334	189.222	148.375	0.009
PFS Q2	3334	195.897	146.769	0.010
PFS Q2	3334	197.963	142.043	0.013
PFS Q2	3334	186.859	154.037	0.009

PFS Q2	3334	212.674	141.077	0.007
PFS Q2	3334	198.518	149.000	0.011
PFS Q2	3334	197.978	143.400	0.010
PFS Q2	3334	193.688	151.565	0.007
PFS Q2	3334	198.827	140.000	0.013
PFS Q2	4445	203.500	139.000	0.004
PFS Q2	4445	202.347	143.043	0.005
PFS Q2	4445	202.094	143.400	0.014
PFS Q2	4445	209.469	149.542	0.005
PFS Q2	4445	207.411	150.000	0.006
PFS Q2	4445	215.558	151.056	0.005
PFS Q2	4445	210.334	144.542	0.004
PFS Q2	4445	204.366	151.063	0.011
PFS Q2	4445	202.357	149.813	0.005
PFS Q2	4445	213.589	151.750	0.008
PFS Q2	5556	215.040	144.077	0.005
PFS Q2	5556	212.250	152.769	0.010
PFS Q2	5556	215.639	154.250	0.006
PFS Q2	5556	207.607	149.462	0.005
PFS Q2	5556	208.186	142.077	0.004
PFS Q2	5556	206.958	155.750	0.011
PFS Q2	5556	220.065	147.077	0.006
PFS Q2	5556	215.773	153.130	0.003
PFS Q2	5556	207.500	148.462	0.005
PFS Q2	5556	216.940	139.125	0.002
PFS Q2	6667	213.200	165.476	0.007
PFS Q2	6667	208.657	165.167	0.004
PFS Q2	6667	210.423	144.120	0.004
PFS Q2	6667	219.109	141.214	0.003
PFS Q2	6667	212.630	148.773	0.004
PFS Q2	6667	207.552	154.556	0.012
PFS Q2	6667	217.600	159.250	0.005
PFS Q2	6667	208.501	143.500	0.004
PFS Q2	6667	216.920	149.773	0.003
PFS Q2	6667	220.980	150.500	0.002
PFS Q2	7778	214.915	160.308	0.039
PFS Q2	7778	219.630	166.833	0.040
PFS Q2	7778	219.257	165.588	0.038
PFS Q2	7778	218.143	159.773	0.041
PFS Q2	7778	221.429	157.545	0.040
PFS Q2	7778	227.800	155.895	0.045
PFS Q2	7778	215.850	160.250	0.054
PFS Q2	7778	214.333	154.600	0.040
PFS Q2	7778	225.136	161.550	0.032
PFS Q2	7778	223.128	154.889	0.055
PFS Q2	8889	230.316	179.111	0.107
PFS Q2	8889	227.107	175.620	0.091
PFS Q2	8889	231.799	179.286	0.103
PFS Q2	8889	230.821	176.094	0.107
PFS Q2	8889	230.607	177.250	0.092
PFS Q2	8889	229.400	179.717	0.102
PFS Q2	8889	231.344	178.071	0.070

PFS Q2	8889	231.903	174.615	0.102
PFS Q2	8889	228.436	178.618	0.109
PFS Q2	8889	233.701	176.176	0.092
PFS Q2	10000	237.167	183.909	0.077
PFS Q2	10000	225.000	178.125	0.123
PFS Q2	10000	233.120	178.000	0.130
PFS Q2	10000	233.966	183.545	0.088
PFS Q2	10000	240.550	182.714	0.120
PFS Q2	10000	235.000	174.652	0.097
PFS Q2	10000	231.600	184.750	0.124
PFS Q2	10000	235.273	184.000	0.114
PFS Q2	10000	236.059	180.125	0.102
PFS Q2	10000	234.091	183.250	0.117
OS Q2	1112	159.238	141.563	0.120
OS Q2	1112	148.720	140.500	0.323
OS Q2	1112	149.063	132.176	0.231
OS Q2	1112	149.386	133.545	0.188
OS Q2	1112	151.882	131.063	0.125
OS Q2	1112	158.038	132.909	0.280
OS Q2	1112	155.271	137.000	0.170
OS Q2	1112	155.158	135.056	0.186
OS Q2	1112	153.028	148.500	0.211
OS Q2	1112	158.823	139.652	0.296
OS Q2	2223	163.909	145.500	0.195
OS Q2	2223	167.019	150.000	0.237
OS Q2	2223	175.533	149.167	0.266
OS Q2	2223	176.094	150.176	0.235
OS Q2	2223	177.318	144.800	0.102
OS Q2	2223	182.819	154.375	0.209
OS Q2	2223	176.038	146.000	0.266
OS Q2	2223	183.100	144.250	0.084
OS Q2	2223	171.497	154.429	0.298
OS Q2	2223	169.881	144.550	0.148
OS Q2	3334	180.027	144.316	0.148
OS Q2	3334	176.713	148.375	0.104
OS Q2	3334	181.333	162.588	0.203
OS Q2	3334	182.032	149.143	0.140
OS Q2	3334	177.298	153.474	0.102
OS Q2	3334	197.083	163.056	0.099
OS Q2	3334	186.031	155.154	0.207
OS Q2	3334	176.563	146.750	0.195
OS Q2	3334	182.186	151.565	0.088
OS Q2	3334	181.143	149.000	0.225
OS Q2	4445	182.221	149.435	0.143
OS Q2	4445	195.957	147.000	0.121
OS Q2	4445	190.936	159.550	0.231
OS Q2	4445	190.488	149.542	0.156
OS Q2	4445	191.876	151.056	0.184
OS Q2	4445	197.124	173.000	0.146
OS Q2	4445	188.193	150.000	0.120
OS Q2	4445	189.177	159.900	0.170
OS Q2	4445	185.789	169.217	0.166

OS Q2	4445	185.036	164.308	0.117
OS Q2	5556	197.032	161.474	0.102
OS Q2	5556	198.357	157.667	0.219
OS Q2	5556	201.283	158.789	0.112
OS Q2	5556	204.337	155.789	0.134
OS Q2	5556	186.407	169.000	0.164
OS Q2	5556	196.710	164.714	0.153
OS Q2	5556	194.696	174.000	0.143
OS Q2	5556	190.639	153.130	0.112
OS Q2	5556	197.607	163.625	0.104
OS Q2	5556	194.356	160.750	0.117
OS Q2	6667	202.121	165.476	0.120
OS Q2	6667	197.984	176.000	0.139
OS Q2	6667	190.617	155.588	0.113
OS Q2	6667	195.115	167.900	0.121
OS Q2	6667	192.359	159.053	0.130
OS Q2	6667	197.221	159.750	0.161
OS Q2	6667	210.678	159.250	0.102
OS Q2	6667	192.923	156.000	0.170
OS Q2	6667	197.896	158.550	0.140
OS Q2	6667	200.286	160.909	0.130
OS Q2	7778	210.065	160.308	0.021
OS Q2	7778	199.875	156.120	0.023
OS Q2	7778	205.900	165.588	0.021
OS Q2	7778	201.625	159.773	0.021
OS Q2	7778	202.106	157.545	0.025
OS Q2	7778	212.438	155.556	0.022
OS Q2	7778	206.850	156.552	0.023
OS Q2	7778	211.228	154.600	0.021
OS Q2	7778	221.475	161.550	0.015
OS Q2	7778	206.316	154.889	0.051
OS Q2	8889	221.191	179.111	0.056
OS Q2	8889	215.896	175.620	0.060
OS Q2	8889	223.575	181.450	0.063
OS Q2	8889	217.578	177.250	0.066
OS Q2	8889	223.769	177.250	0.052
OS Q2	8889	214.892	179.717	0.059
OS Q2	8889	225.968	178.071	0.038
OS Q2	8889	228.533	174.615	0.053
OS Q2	8889	222.636	178.618	0.064
OS Q2	8889	220.824	176.176	0.056
OS Q2	10000	236.050	184.556	0.045
OS Q2	10000	225.000	180.000	0.067
OS Q2	10000	219.550	178.000	0.055
OS Q2	10000	233.833	193.120	0.031
OS Q2	10000	240.273	188.545	0.045
OS Q2	10000	224.542	174.652	0.053
OS Q2	10000	224.667	196.235	0.058
OS Q2	10000	228.040	184.000	0.050
OS Q2	10000	224.955	180.125	0.059
OS Q2	10000	232.000	195.077	0.047
Age Q3	1112	131.000	160.000	0.038

Age Q3	1112	123.023	153.000	0.049
Age Q3	1112	126.498	149.071	0.037
Age Q3	1112	130.250	148.167	0.076
Age Q3	1112	133.733	150.714	0.091
Age Q3	1112	130.975	152.545	0.076
Age Q3	1112	119.071	152.036	0.020
Age Q3	1112	125.694	153.316	0.102
Age Q3	1112	121.500	154.000	0.056
Age Q3	1112	126.294	158.333	0.042
Age Q3	2223	136.375	164.484	0.051
Age Q3	2223	138.903	168.037	0.048
Age Q3	2223	135.371	176.000	0.041
Age Q3	2223	139.771	170.545	0.054
Age Q3	2223	129.080	172.500	0.035
Age Q3	2223	127.083	182.750	0.020
Age Q3	2223	128.994	174.000	0.070
Age Q3	2223	136.125	182.045	0.068
Age Q3	2223	132.261	167.538	0.051
Age Q3	2223	137.353	173.400	0.032
Age Q3	3334	134.273	174.714	0.041
Age Q3	3334	136.176	176.250	0.029
Age Q3	3334	137.955	176.000	0.043
Age Q3	3334	134.172	179.231	0.031
Age Q3	3334	135.750	173.750	0.043
Age Q3	3334	135.538	186.120	0.014
Age Q3	3334	138.333	192.250	0.039
Age Q3	3334	133.940	179.913	0.034
Age Q3	3334	133.949	176.333	0.028
Age Q3	3334	132.000	186.261	0.041
Age Q3	4445	133.100	184.158	0.023
Age Q3	4445	135.381	183.714	0.035
Age Q3	4445	136.867	184.889	0.029
Age Q3	4445	134.200	183.833	0.029
Age Q3	4445	137.571	185.800	0.045
Age Q3	4445	138.333	181.773	0.039
Age Q3	4445	137.278	188.273	0.019
Age Q3	4445	134.833	194.136	0.028
Age Q3	4445	137.563	181.435	0.034
Age Q3	4445	135.469	184.667	0.026
Age Q3	5556	137.138	187.107	0.041
Age Q3	5556	138.250	195.640	0.034
Age Q3	5556	136.833	188.565	0.017
Age Q3	5556	137.583	200.690	0.024
Age Q3	5556	135.705	190.577	0.016
Age Q3	5556	136.033	190.300	0.037
Age Q3	5556	138.125	185.000	0.032
Age Q3	5556	132.690	186.652	0.025
Age Q3	5556	137.667	191.038	0.033
Age Q3	5556	134.563	191.097	0.021
Age Q3	6667	137.767	193.000	0.023
Age Q3	6667	138.000	194.500	0.023
Age Q3	6667	135.833	181.400	0.024

Age Q3	6667	135.774	187.120	0.022
Age Q3	6667	135.118	189.500	0.023
Age Q3	6667	138.125	198.682	0.031
Age Q3	6667	136.167	203.000	0.015
Age Q3	6667	136.700	190.556	0.028
Age Q3	6667	137.958	186.500	0.024
Age Q3	6667	138.000	189.091	0.025
Age Q3	7778	149.875	196.063	0.277
Age Q3	7778	146.615	189.125	0.329
Age Q3	7778	151.000	192.105	0.285
Age Q3	7778	149.955	194.588	0.338
Age Q3	7778	149.333	187.895	0.323
Age Q3	7778	143.607	192.545	0.280
Age Q3	7778	148.875	186.929	0.352
Age Q3	7778	145.500	197.000	0.344
Age Q3	7778	145.464	190.913	0.326
Age Q3	7778	146.115	205.091	0.332
Age Q3	8889	155.875	207.240	0.154
Age Q3	8889	160.143	205.036	0.105
Age Q3	8889	158.000	199.750	0.112
Age Q3	8889	156.400	203.750	0.094
Age Q3	8889	157.769	198.345	0.117
Age Q3	8889	158.143	206.607	0.111
Age Q3	8889	157.000	209.250	0.154
Age Q3	8889	157.769	194.625	0.111
Age Q3	8889	156.667	216.182	0.123
Age Q3	8889	154.000	203.938	0.121
Age Q3	10000	182.845	195.908	0.062
Age Q3	10000	187.371	209.881	0.065
Age Q3	10000	189.013	214.947	0.053
Age Q3	10000	185.741	216.321	0.105
Age Q3	10000	179.571	207.149	0.084
Age Q3	10000	187.323	206.552	0.074
Age Q3	10000	185.851	209.667	0.067
Age Q3	10000	184.060	212.267	0.079
Age Q3	10000	184.211	204.084	0.080
Age Q3	10000	180.125	205.063	0.046
PFS Q3	1112	169.363	141.563	0.054
PFS Q3	1112	159.448	140.500	0.166
PFS Q3	1112	149.063	132.250	0.166
PFS Q3	1112	149.281	139.000	0.204
PFS Q3	1112	185.075	136.462	0.026
PFS Q3	1112	147.588	144.063	0.282
PFS Q3	1112	167.528	137.000	0.054
PFS Q3	1112	151.711	135.647	0.117
PFS Q3	1112	179.303	148.500	0.053
PFS Q3	1112	170.618	139.652	0.068
PFS Q3	2223	183.784	145.500	0.081
PFS Q3	2223	190.957	150.000	0.099
PFS Q3	2223	187.364	149.167	0.081
PFS Q3	2223	181.164	150.176	0.058
PFS Q3	2223	199.156	144.800	0.030

PFS Q3	2223	184.816	154.375	0.117
PFS Q3	2223	194.826	146.000	0.108
PFS Q3	2223	216.269	148.429	0.054
PFS Q3	2223	198.530	154.429	0.074
PFS Q3	2223	175.958	144.550	0.090
PFS Q3	3334	201.506	149.154	0.069
PFS Q3	3334	199.222	150.053	0.061
PFS Q3	3334	186.583	162.588	0.053
PFS Q3	3334	225.003	149.143	0.032
PFS Q3	3334	203.315	161.214	0.058
PFS Q3	3334	203.768	163.056	0.043
PFS Q3	3334	193.563	155.154	0.111
PFS Q3	3334	191.478	154.273	0.088
PFS Q3	3334	209.326	151.714	0.038
PFS Q3	3334	211.667	149.000	0.074
PFS Q3	4445	201.249	149.435	0.039
PFS Q3	4445	210.625	147.000	0.046
PFS Q3	4445	202.094	159.550	0.064
PFS Q3	4445	220.504	153.000	0.041
PFS Q3	4445	212.463	151.056	0.048
PFS Q3	4445	206.058	156.000	0.032
PFS Q3	4445	208.310	172.600	0.042
PFS Q3	4445	205.168	159.900	0.060
PFS Q3	4445	213.045	169.217	0.034
PFS Q3	4445	211.037	164.308	0.056
PFS Q3	5556	215.180	161.474	0.031
PFS Q3	5556	205.607	157.667	0.054
PFS Q3	5556	217.488	158.789	0.051
PFS Q3	5556	205.457	155.789	0.045
PFS Q3	5556	216.450	169.000	0.053
PFS Q3	5556	209.247	164.714	0.039
PFS Q3	5556	217.470	174.000	0.054
PFS Q3	5556	206.107	173.500	0.032
PFS Q3	5556	207.500	163.625	0.035
PFS Q3	5556	218.034	160.750	0.020
PFS Q3	6667	220.058	165.526	0.049
PFS Q3	6667	219.749	176.000	0.025
PFS Q3	6667	210.423	155.588	0.041
PFS Q3	6667	225.863	167.900	0.030
PFS Q3	6667	216.750	159.053	0.026
PFS Q3	6667	198.179	159.750	0.071
PFS Q3	6667	214.500	161.870	0.039
PFS Q3	6667	207.268	154.500	0.037
PFS Q3	6667	221.745	158.550	0.034
PFS Q3	6667	223.065	158.542	0.019
PFS Q3	7778	211.315	174.000	0.123
PFS Q3	7778	219.630	175.500	0.105
PFS Q3	7778	220.859	172.588	0.094
PFS Q3	7778	221.250	174.000	0.086
PFS Q3	7778	221.429	173.130	0.105
PFS Q3	7778	219.893	174.000	0.096
PFS Q3	7778	211.000	172.452	0.102

PFS Q3	7778	211.895	174.000	0.120
PFS Q3	7778	214.136	174.000	0.088
PFS Q3	7778	223.128	174.000	0.128
PFS Q3	8889	255.409	184.688	0.021
PFS Q3	8889	254.524	183.158	0.013
PFS Q3	8889	243.799	186.736	0.018
PFS Q3	8889	248.514	179.344	0.014
PFS Q3	8889	256.774	183.542	0.016
PFS Q3	8889	253.242	186.717	0.015
PFS Q3	8889	253.209	183.271	0.013
PFS Q3	8889	249.013	180.365	0.019
PFS Q3	8889	252.828	186.156	0.025
PFS Q3	8889	254.341	180.226	0.017
PFS Q3	10000	249.413	187.640	0.043
PFS Q3	10000	243.833	187.650	0.057
PFS Q3	10000	261.871	188.778	0.042
PFS Q3	10000	248.517	202.560	0.040
PFS Q3	10000	256.999	194.828	0.068
PFS Q3	10000	245.786	185.563	0.049
PFS Q3	10000	249.550	199.656	0.065
PFS Q3	10000	250.747	186.976	0.054
PFS Q3	10000	241.448	187.320	0.073
PFS Q3	10000	247.045	196.104	0.066
OS Q3	1112	141.487	152.667	0.440
OS Q3	1112	138.273	141.429	0.277
OS Q3	1112	151.475	139.500	0.388
OS Q3	1112	156.854	139.000	0.422
OS Q3	1112	155.200	137.647	0.465
OS Q3	1112	160.074	138.615	0.416
OS Q3	1112	136.589	145.636	0.491
OS Q3	1112	153.554	136.400	0.400
OS Q3	1112	150.900	152.056	0.403
OS Q3	1112	143.950	140.154	0.494
OS Q3	2223	158.428	149.000	0.409
OS Q3	2223	168.132	165.750	0.484
OS Q3	2223	165.034	156.050	0.491
OS Q3	2223	171.635	155.250	0.465
OS Q3	2223	173.162	167.286	0.440
OS Q3	2223	171.911	159.786	0.465
OS Q3	2223	162.528	153.200	0.422
OS Q3	2223	184.393	162.789	0.315
OS Q3	2223	158.727	158.250	0.497
OS Q3	2223	160.531	154.391	0.465
OS Q3	3334	171.445	169.038	0.434
OS Q3	3334	171.026	168.053	0.352
OS Q3	3334	178.571	164.792	0.422
OS Q3	3334	165.865	162.120	0.497
OS Q3	3334	175.609	161.800	0.321
OS Q3	3334	177.919	164.636	0.391
OS Q3	3334	180.608	172.231	0.397
OS Q3	3334	175.822	173.200	0.459
OS Q3	3334	157.084	167.565	0.484

OS Q3	3334	173.393	174.217	0.428
OS Q3	4445	183.101	173.750	0.367
OS Q3	4445	174.785	172.000	0.352
OS Q3	4445	181.435	172.619	0.428
OS Q3	4445	183.635	173.000	0.422
OS Q3	4445	188.250	176.000	0.478
OS Q3	4445	192.339	175.000	0.416
OS Q3	4445	177.713	180.667	0.394
OS Q3	4445	183.910	174.875	0.453
OS Q3	4445	172.101	173.000	0.447
OS Q3	4445	182.565	168.240	0.440
OS Q3	5556	187.227	177.500	0.370
OS Q3	5556	187.019	177.000	0.391
OS Q3	5556	192.342	174.250	0.376
OS Q3	5556	190.450	174.600	0.361
OS Q3	5556	177.939	174.588	0.447
OS Q3	5556	180.178	172.000	0.494
OS Q3	5556	189.565	177.103	0.358
OS Q3	5556	187.880	174.474	0.422
OS Q3	5556	185.043	174.000	0.307
OS Q3	5556	189.088	177.368	0.347
OS Q3	6667	187.436	181.368	0.419
OS Q3	6667	183.436	181.176	0.447
OS Q3	6667	183.007	175.917	0.434
OS Q3	6667	194.750	179.231	0.361
OS Q3	6667	190.454	174.333	0.400
OS Q3	6667	186.177	174.333	0.382
OS Q3	6667	187.250	175.037	0.409
OS Q3	6667	182.341	181.625	0.413
OS Q3	6667	188.482	179.474	0.434
OS Q3	6667	186.105	181.375	0.406
OS Q3	7778	227.067	175.345	0.028
OS Q3	7778	214.800	180.955	0.054
OS Q3	7778	226.114	174.000	0.037
OS Q3	7778	220.761	182.897	0.040
OS Q3	7778	229.317	174.000	0.035
OS Q3	7778	240.626	177.476	0.036
OS Q3	7778	221.052	174.000	0.047
OS Q3	7778	221.533	183.895	0.041
OS Q3	7778	239.433	181.000	0.029
OS Q3	7778	230.042	185.929	0.044
OS Q3	8889	240.391	186.556	0.067
OS Q3	8889	243.650	190.750	0.063
OS Q3	8889	234.885	188.900	0.056
OS Q3	8889	237.391	191.500	0.085
OS Q3	8889	248.667	191.607	0.054
OS Q3	8889	239.000	192.500	0.059
OS Q3	8889	240.773	192.000	0.061
OS Q3	8889	234.897	189.130	0.054
OS Q3	8889	232.500	195.200	0.054
OS Q3	8889	239.526	190.526	0.052
OS Q3	10000	239.125	192.895	0.117

OS Q3	10000	241.778	196.037	0.143
OS Q3	10000	239.300	202.111	0.126
OS Q3	10000	258.000	212.000	0.092
OS Q3	10000	240.550	199.000	0.120
OS Q3	10000	242.550	197.370	0.094
OS Q3	10000	241.316	207.769	0.136
OS Q3	10000	237.885	193.000	0.140
OS Q3	10000	253.667	191.000	0.128
OS Q3	10000	242.500	197.235	0.140

Table S2. Association of bacterial species abundance values at phyllum level with progression free survival (PFS) and overall survival (OS) values. Log-rank test was applied. P-values are showed without adjustment for multiple comparisons.

Characteristics	Taxa	Threshold	Size <	Size >=	Log-rank p-value
PFS	Fusicatenibacter	0.002	29	34	0.001
PFS	Faecalibacterium	0.033	32	31	0.001
PFS	Oscillibacter	0.002	46	17	0.002
PFS	Lachnospiraceae	0.006	16	47	0.002
PFS	Dorea	0.001	17	46	0.006
PFS	[Eubacterium] hallii group	0.002	49	14	0.009
PFS	Ruminococcaceae NK4A214 group	0.002	25	38	0.016
PFS	Lachnospira	0.002	32	31	0.016
PFS	Ruminococcaceae NK4A214 group	0.006	49	14	0.022
PFS	Lachnospiraceae ND3007 group	0.001	38	25	0.026
PFS	Sutterella	0.002	48	15	0.028
PFS	Ruminococcaceae UCG-002	0.014	16	47	0.031
PFS	Methanobrevibacter	0.005	34	29	0.032
PFS	Coprococcus 2	0.004	45	18	0.032
PFS	Ruminococcus 2	0.003	17	46	0.034
PFS	Collinsella	0.009	32	31	0.037
PFS	Dialister	0.009	31	32	0.038
PFS	[Eubacterium] eligens group	0.01	47	16	0.041
PFS	Ruminococcus 2	0.011	32	31	0.045
PFS	Collinsella	0.001	16	47	0.049
PFS	Family XIII AD3011 group	0.002	48	15	0.053
PFS	Lachnoclostridium	0.001	26	37	0.060
PFS	Ruminococcaceae NK4A214 group	0.001	18	45	0.060
PFS	Faecalibacterium	0.021	16	47	0.063
PFS	Subdoligranulum	0.011	19	44	0.066
PFS	Collinsella	0.026	47	16	0.067
PFS	Alistipes	0.048	47	16	0.076
PFS	Bifidobacterium	0.011	32	31	0.076
PFS	Ruminococcaceae UCG-003	0.001	33	30	0.077
PFS	Akkermansia	0.037	47	16	0.078
PFS	Prevotella 7	0.001	48	15	0.080
PFS	Coprococcus 3	0.001	19	44	0.080
PFS	Roseburia	0.003	13	50	0.094
PFS	Ruminococcaceae UCG-014	0.01	31	32	0.100
PFS	Bacteroides	0.126	32	31	0.106
PFS	Barnesiella	0.012	46	17	0.108
PFS	[Eubacterium] eligens group	0.004	34	29	0.113
PFS	Faecalibacterium	0.06	47	16	0.124
PFS	Eggerthellaceae uncultured	0.002	45	18	0.125

PFS	Howardella	0.001	43	20	0.136
PFS	Prevotella 9	0.004	32	31	0.138
PFS	Erysipelotrichaceae UCG-003	0.003	46	17	0.143
PFS	Blautia	0.002	17	46	0.144
PFS	Christensenellaceae R-7 group	0.002	18	45	0.147
PFS	Mollicutes RF39 uncultured bacterium	0.004	48	15	0.149
PFS	Coprococcus 2	0.001	31	32	0.150
PFS	UBA1819	0.003	49	14	0.165
PFS	Family XIII AD3011 group	0.001	40	23	0.169
PFS	Clostridium sensu stricto 1	0.001	42	21	0.170
PFS	Senegalimassilia	0.004	48	15	0.173
PFS	Streptococcus	0.002	16	47	0.174
PFS	Ruminococcaceae UCG-002	0.026	32	31	0.195
PFS	Ruminococcaceae UCG-013	0.001	28	35	0.196
PFS	Butyricimonas	0.007	46	17	0.204
PFS	Lachnospiraceae NK4A136 group	0.001	15	48	0.205
PFS	Coprococcus 1	0.001	51	12	0.212
PFS	Lachnospiraceae uncultured	0.001	27	36	0.274
PFS	Ruminococcaceae UCG-014	0.032	47	16	0.286
PFS	Anaerostipes	0.001	49	14	0.287
PFS	Romboutsia	0.001	45	18	0.290
PFS	Veillonella	0.003	47	16	0.293
PFS	Dorea	0.004	37	26	0.307
PFS	Methanobrevibacter	0.023	47	16	0.311
PFS	Unassigned	0.002	34	29	0.316
PFS	Lachnospiraceae uncultured	0.003	44	19	0.341
PFS	Barnesiella	0.003	31	32	0.345
PFS	Lactobacillus	0.003	47	16	0.358
PFS	Negativibacillus	0.001	52	11	0.366
PFS	[Eubacterium] hallii group	0.001	36	27	0.373
PFS	Bilophila	0.002	48	15	0.376
PFS	Lachnospiraceae	0.024	47	16	0.389
PFS	Paraprevotella	0.001	31	32	0.391
PFS	Ruminococcus 1	0.004	34	29	0.392
PFS	Ruminococcaceae UCG-002	0.042	46	17	0.393
PFS	Alistipes	0.031	32	31	0.395
PFS	Lachnospiraceae NK4A136 group	0.011	45	18	0.399
PFS	Prevotella 2	0.002	47	16	0.399
PFS	[Eubacterium] coprostanoligenes group	0.027	47	16	0.400
PFS	Slackia	0.002	45	18	0.402
PFS	Coprococcus 3	0.006	45	18	0.411
PFS	Ruminococcaceae UCG-003	0.004	49	14	0.440
PFS	Roseburia	0.014	47	16	0.440
PFS	Dorea	0.005	45	18	0.443
PFS	Ruminiclostridium 5	0.001	31	32	0.446
PFS	Ruminiclostridium 5	0.002	41	22	0.449
PFS	Prevotellaceae uncultured	0.001	47	16	0.461
PFS	Subdoligranulum	0.026	47	16	0.464
PFS	Catenibacterium	0.01	46	17	0.468
PFS	Fusicatenibacter	0.006	46	17	0.484
PFS	Escherichia-Shigella	0.002	15	48	0.486
PFS	Escherichia-Shigella	0.03	48	15	0.487
PFS	Ruminiclostridium 6	0.004	46	17	0.489
PFS	[Ruminococcus] torques group	0.002	14	49	0.489
PFS	Butyricimonas	0.002	33	30	0.495

PFS	Butyricicoccus	0.001	38	25	0.507
PFS	Lachnospira	0.006	47	16	0.519
PFS	Streptococcus	0.021	47	16	0.524
PFS	Lachnospiraceae	0.012	32	31	0.542
PFS	Parabacteroides	0.022	49	14	0.547
PFS	Ruminococcaceae uncultured	0.009	30	33	0.550
PFS	Lachnospiraceae ND3007 group	0.002	50	13	0.554
PFS	Ruminococcaceae UCG-005	0.006	31	32	0.576
PFS	Subdoligranulum	0.017	32	31	0.576
PFS	[Eubacterium] coprostanoligenes group	0.006	16	47	0.579
PFS	Parabacteroides	0.01	33	30	0.590
PFS	Coprobacter	0.001	49	14	0.593
PFS	Clostridiales vadinBB60 group uncultured bacterium	0.004	47	16	0.593
PFS	Parabacteroides	0.002	17	46	0.597
PFS	[Eubacterium] coprostanoligenes group	0.017	31	32	0.597
PFS	Christensenellaceae R-7 group	0.019	47	16	0.600
PFS	Butyricicoccus	0.002	51	12	0.614
PFS	Solobacterium	0.002	47	16	0.621
PFS	Christensenellaceae R-7 group	0.009	34	29	0.621
PFS	Ruminococcus 2	0.019	47	16	0.627
PFS	Desulfovibrio	0.006	46	17	0.628
PFS	Ruminococcaceae uncultured	0.004	16	47	0.640
PFS	Ruminococcaceae UCG-013	0.004	47	16	0.641
PFS	Muribaculaceae uncultured bacterium	0.004	32	31	0.649
PFS	Ruminococcaceae UCG-010	0.005	46	17	0.650
PFS	Holdemanella	0.008	47	16	0.654
PFS	Akkermansia	0.002	32	31	0.666
PFS	Dialister	0.028	47	16	0.669
PFS	Parasutterella	0.002	48	15	0.670
PFS	Ruminiclostridium 9	0.001	38	25	0.672
PFS	Ruminiclostridium 9	0.001	38	25	0.672
PFS	Lactobacillus	0.001	40	23	0.691
PFS	Ruminococcus 1	0.009	48	15	0.699
PFS	[Ruminococcus] torques group	0.01	45	18	0.701
PFS	Unassigned	0.001	23	40	0.703
PFS	[Ruminococcus] torques group	0.006	30	33	0.704
PFS	Paraprevotella	0.007	48	15	0.715
PFS	Rikenellaceae RC9 gut group	0.008	47	16	0.717
PFS	Escherichia-Shigella	0.008	31	32	0.728
PFS	Phascolarctobacterium	0.008	47	16	0.737
PFS	Ruminococcaceae UCG-005	0.002	15	48	0.747
PFS	Klebsiella	0.002	45	18	0.751
PFS	Rothia	0.001	47	16	0.773
PFS	Roseburia	0.006	30	33	0.775
PFS	Lachnoclostridium	0.004	45	18	0.776
PFS	Bifidobacterium	0.026	47	16	0.787
PFS	Lachnospiraceae NK4A136 group	0.003	29	34	0.792
PFS	Odoribacter	0.003	30	33	0.806
PFS	Odoribacter	0.007	48	15	0.809
PFS	Lachnospiraceae UCG-001	0.001	51	12	0.812
PFS	[Eubacterium] xylanophilum group	0.003	52	11	0.818
PFS	Enterorhabdus	0.002	51	12	0.828
PFS	Bacteroides	0.25	47	16	0.834
PFS	[Eubacterium] ruminantium group	0.001	50	13	0.837

PFS	Alistipes	0.008	16	47	0.844
PFS	Turicibacter	0.001	51	12	0.849
PFS	Coprococcus 3	0.003	34	29	0.856
PFS	Haemophilus	0.001	50	13	0.872
PFS	Actinomyces	0.001	44	19	0.886
PFS	Alloprevotella	0.007	47	16	0.887
PFS	CAG-56	0.001	48	15	0.902
PFS	Ruminococcaceae UCG-005	0.011	46	17	0.928
PFS	Ruminococcus 1	0.001	14	49	0.934
PFS	Blautia	0.004	36	27	0.937
PFS	Unassigned	0.006	47	16	0.945
PFS	Blautia	0.007	46	17	0.947
PFS	Lactococcus	0.001	48	15	0.947
PFS	Prevotella 9	0.051	47	16	0.952
PFS	Bacteroides	0.078	17	46	0.970
PFS	Ruminococcaceae uncultured	0.015	48	15	0.971
PFS	Erysipelatoclostridium	0.001	47	16	0.982
PFS	Muribaculaceae uncultured bacterium	0.012	47	16	0.984
PFS	Ruminococcaceae UCG-010	0.002	31	32	0.985
PFS	[Eubacterium] ventriosum group	0.001	52	11	0.987
PFS	Streptococcus	0.006	29	34	0.997
OS	[Eubacterium] hallii group	0.002	49	14	0.001
OS	Prevotella 7	0.001	48	15	0.021
OS	Slackia	0.002	45	18	0.028
OS	Streptococcus	0.006	29	34	0.030
OS	Alistipes	0.048	47	16	0.042
OS	Lachnospiraceae UCG-001	0.001	51	12	0.045
OS	Butyricimonas	0.007	46	17	0.062
OS	Blautia	0.004	36	27	0.094
OS	Coprobacter	0.001	49	14	0.095
OS	Ruminococcus 1	0.009	48	15	0.097
OS	[Eubacterium] xylanophilum group	0.003	52	11	0.097
OS	Parabacteroides	0.022	49	14	0.098
OS	Ruminococcaceae NK4A214 group	0.002	25	38	0.102
OS	Fusicatenibacter	0.006	46	17	0.109
OS	Family XIII AD3011 group	0.002	48	15	0.109
OS	Odoribacter	0.007	48	15	0.118
OS	Muribaculaceae uncultured bacterium	0.012	47	16	0.119
OS	Ruminococcaceae NK4A214 group	0.006	49	14	0.121
OS	Coprococcus 3	0.006	45	18	0.121
OS	Bifidobacterium	0.011	32	31	0.123
OS	Lachnospiraceae	0.006	16	47	0.125
OS	Methanobrevibacter	0.005	34	29	0.126
OS	Senegalimassilia	0.004	48	15	0.126
OS	Sutterella	0.002	48	15	0.133
OS	Lachnospiraceae uncultured	0.003	44	19	0.138
OS	Ruminococcaceae NK4A214 group	0.001	18	45	0.139
OS	Ruminococcaceae UCG-002	0.014	16	47	0.149
OS	Veillonella	0.003	47	16	0.150
OS	Ruminococcaceae UCG-003	0.001	33	30	0.150
OS	Anaerostipes	0.001	49	14	0.154
OS	Oscillibacter	0.002	46	17	0.155
OS	Prevotella 9	0.004	32	31	0.173
OS	Clostridium sensu stricto 1	0.001	42	21	0.175
OS	Akkermansia	0.037	47	16	0.177

OS	Erysipelotrichaceae UCG-003	0.003	46	17	0.189
OS	[Eubacterium] eligens group	0.01	47	16	0.191
OS	Alistipes	0.031	32	31	0.191
OS	Dorea	0.005	45	18	0.192
OS	Roseburia	0.003	13	50	0.199
OS	Butyricimonas	0.002	33	30	0.206
OS	Howardella	0.001	43	20	0.206
OS	Mollicutes RF39 uncultured bacterium	0.004	48	15	0.210
OS	Barnesiella	0.012	46	17	0.211
OS	Blautia	0.007	46	17	0.217
OS	Coprococcus 1	0.001	51	12	0.218
OS	Dorea	0.004	37	26	0.220
OS	Ruminiclostridium 5	0.001	31	32	0.232
OS	UBA1819	0.003	49	14	0.233
OS	[Eubacterium] hallii group	0.001	36	27	0.233
OS	Streptococcus	0.021	47	16	0.253
OS	Coprococcus 3	0.003	34	29	0.259
OS	Streptococcus	0.002	16	47	0.261
OS	Bifidobacterium	0.026	47	16	0.262
OS	Dialister	0.009	31	32	0.267
OS	Paraprevotella	0.001	31	32	0.273
OS	Holdemanella	0.008	47	16	0.275
OS	Coprococcus 2	0.004	45	18	0.278
OS	Coprococcus 2	0.001	31	32	0.278
OS	Bacteroides	0.126	32	31	0.283
OS	Eggerthellaceae uncultured	0.002	45	18	0.288
OS	Alistipes	0.008	16	47	0.292
OS	Actinomyces	0.001	44	19	0.301
OS	Ruminococcaceae UCG-013	0.004	47	16	0.309
OS	Faecalibacterium	0.06	47	16	0.311
OS	Escherichia-Shigella	0.008	31	32	0.315
OS	Ruminiclostridium 5	0.002	41	22	0.321
OS	Methanobrevibacter	0.023	47	16	0.336
OS	Turicibacter	0.001	51	12	0.349
OS	Subdoligranulum	0.011	19	44	0.350
OS	Haemophilus	0.001	50	13	0.357
OS	Lachnoclostridium	0.001	26	37	0.361
OS	Ruminococcaceae UCG-002	0.042	46	17	0.369
OS	Subdoligranulum	0.026	47	16	0.371
OS	[Ruminococcus] torques group	0.002	14	49	0.375
OS	[Ruminococcus] torques group	0.006	30	33	0.379
OS	[Eubacterium] ventriosum group	0.001	52	11	0.384
OS	Ruminococcus 1	0.004	34	29	0.389
OS	Ruminiclostridium 6	0.004	46	17	0.395
OS	Blautia	0.002	17	46	0.406
OS	Lachnospiraceae	0.024	47	16	0.433
OS	Lachnoclostridium	0.004	45	18	0.436
OS	Ruminococcus 2	0.011	32	31	0.437
OS	Rothia	0.001	47	16	0.443
OS	Solobacterium	0.002	47	16	0.447
OS	Alloprevotella	0.007	47	16	0.454
OS	Ruminiclostridium 9	0.001	38	25	0.459
OS	Ruminiclostridium 9	0.001	38	25	0.459
OS	Parasutterella	0.002	48	15	0.461
OS	Collinsella	0.009	32	31	0.465

OS	Prevotella 9	0.051	47	16	0.469
OS	Ruminococcus 2	0.019	47	16	0.483
OS	Desulfovibrio	0.006	46	17	0.486
OS	Ruminococcus 1	0.001	14	49	0.492
OS	Dialister	0.028	47	16	0.497
OS	Lachnospiraceae uncultured	0.001	27	36	0.500
OS	Christensenellaceae R-7 group	0.019	47	16	0.519
OS	Ruminococcus 2	0.003	17	46	0.527
OS	Ruminococcaceae uncultured	0.015	48	15	0.528
OS	Lachnospira	0.006	47	16	0.529
OS	Parabacteroides	0.01	33	30	0.533
OS	Faecalibacterium	0.033	32	31	0.538
OS	Enterorhabdus	0.002	51	12	0.547
OS	Lachnospira	0.002	32	31	0.548
OS	Negativibacillus	0.001	52	11	0.557
OS	Prevotellaceae uncultured	0.001	47	16	0.559
OS	Lactococcus	0.001	48	15	0.559
OS	Ruminococcaceae UCG-005	0.006	31	32	0.559
OS	Fusicatenibacter	0.002	29	34	0.560
OS	Roseburia	0.014	47	16	0.564
OS	Dorea	0.001	17	46	0.568
OS	Unassigned	0.002	34	29	0.573
OS	Clostridiales vadinBB60 group uncultured bacterium	0.004	47	16	0.577
OS	Rikenellaceae RC9 gut group	0.008	47	16	0.580
OS	Bacteroides	0.25	47	16	0.582
OS	Unassigned	0.001	23	40	0.589
OS	Christensenellaceae R-7 group	0.002	18	45	0.593
OS	Coprococcus 3	0.001	19	44	0.595
OS	Catenibacterium	0.01	46	17	0.597
OS	Escherichia-Shigella	0.002	15	48	0.601
OS	[Eubacterium] ruminantium group	0.001	50	13	0.602
OS	Butyricicoccus	0.002	51	12	0.606
OS	Family XIII AD3011 group	0.001	40	23	0.615
OS	Prevotella 2	0.002	47	16	0.631
OS	Roseburia	0.006	30	33	0.633
OS	Ruminococcaceae uncultured	0.004	16	47	0.637
OS	Phascolarctobacterium	0.008	47	16	0.645
OS	Lactobacillus	0.001	40	23	0.653
OS	Collinsella	0.026	47	16	0.662
OS	Ruminococcaceae UCG-002	0.026	32	31	0.664
OS	Ruminococcaceae UCG-005	0.002	15	48	0.666
OS	Muribaculaceae uncultured bacterium	0.004	32	31	0.667
OS	Barnesiella	0.003	31	32	0.671
OS	Ruminococcaceae uncultured	0.009	30	33	0.677
OS	Paraprevotella	0.007	48	15	0.677
OS	Romboutsia	0.001	45	18	0.690
OS	Butyricicoccus	0.001	38	25	0.694
OS	Collinsella	0.001	16	47	0.709
OS	Ruminococcaceae UCG-014	0.01	31	32	0.728
OS	Erysipelatoclostridium	0.001	47	16	0.732
OS	Ruminococcaceae UCG-013	0.001	28	35	0.735
OS	[Eubacterium] coprostanoligenes group	0.017	31	32	0.754
OS	Ruminococcaceae UCG-010	0.002	31	32	0.760
OS	Ruminococcaceae UCG-014	0.032	47	16	0.779

OS	Ruminococcaceae UCG-005	0.011	46	17	0.780
OS	Lachnospiraceae ND3007 group	0.002	50	13	0.786
OS	Ruminococcaceae UCG-003	0.004	49	14	0.786
OS	Escherichia-Shigella	0.03	48	15	0.797
OS	[Eubacterium] eligens group	0.004	34	29	0.807
OS	[Eubacterium] coprostanoligenes group	0.006	16	47	0.845
OS	[Ruminococcus] torques group	0.01	45	18	0.862
OS	Bilophila	0.002	48	15	0.874
OS	Lactobacillus	0.003	47	16	0.880
OS	Lachnospiraceae	0.012	32	31	0.880
OS	CAG-56	0.001	48	15	0.885
OS	Bacteroides	0.078	17	46	0.895
OS	[Eubacterium] coprostanoligenes group	0.027	47	16	0.896
OS	Lachnospiraceae NK4A136 group	0.003	29	34	0.897
OS	Subdoligranulum	0.017	32	31	0.899
OS	Klebsiella	0.002	45	18	0.926
OS	Lachnospiraceae NK4A136 group	0.001	15	48	0.927
OS	Christensenellaceae R-7 group	0.009	34	29	0.933
OS	Lachnospiraceae NK4A136 group	0.011	45	18	0.937
OS	Odoribacter	0.003	30	33	0.946
OS	Ruminococcaceae UCG-010	0.005	46	17	0.947
OS	Unassigned	0.006	47	16	0.960
OS	Akkermansia	0.002	32	31	0.967
OS	Parabacteroides	0.002	17	46	0.975
OS	Lachnospiraceae ND3007 group	0.001	38	25	0.983
OS	Faecalibacterium	0.021	16	47	0.999
